# A Challenging Case of Limb Salvage Requiring a Combination of Composite Fixation and Masquelet Technique to Address Significant Segmental Bone Loss

**DOI:** 10.1155/2015/369469

**Published:** 2015-02-19

**Authors:** C. Blair, A. T. Simela, B. J. Cross

**Affiliations:** ^1^Department of Orthopedic Surgery, Broward Health Medical Center, Fort Lauderdale, FL, USA; ^2^University Hospitals, Richmond Medical Center, Cleveland, OH, USA

## Abstract

Cases of limb salvage following skeletal trauma involving significant bone loss pose a particular challenge to the reconstructive surgeon. Certain techniques for addressing this complex issue have been advanced in recent years and have met with considerable success. The Masquelet technique involves a staged procedure in which a temporary skeletal stabilization is paired with implantation of an antibiotic spacer and left in place for 6–8 weeks, during which time a “pseudomembrane” forms around the cement spacer. During the second stage of the procedure, the pseudomembrane is incised, the antibiotic spacer removed, and bone graft is placed. We present a case of significant segmental femur loss in a 19-year-old male opting for limb salvage in which a 17-centimeter segmental loss of bone was essentially regrown using a combination of the Masquelet technique with supplemental endosteal fixation.

## 1. Introduction

Both the Masquelet technique and composite fixation have been proven successful at overcoming segmental bone loss. We hypothesized that when used together, the combined characteristics of these two techniques could effectively provide superior construct stability as well as acting a conduit for new bone growth in a particularly challenging case of segmental bone loss in an open femur fracture with retained distal neurovascular functionality.

## 2. Case Presentation

**Figure 1:**
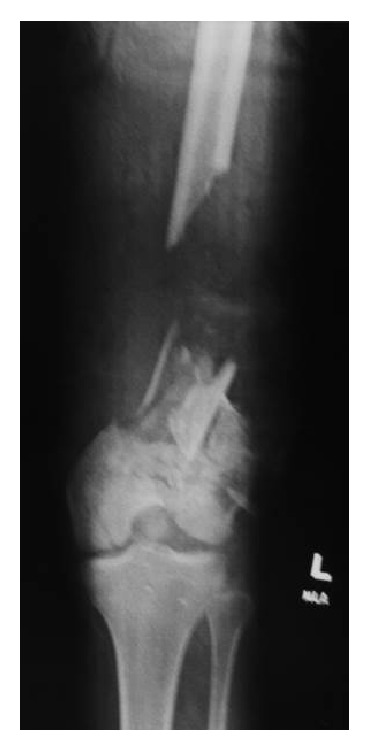
Original injury depicting 17 cm of segmental bone loss in the distal metadiaphyseal aspect of the right femur.

A 19-year-old male patient, injured as a result of a single vehicle motorcycle accident, was accepted as a trauma transfer to our Level I trauma center for definitive evaluation and management of an open femur fracture with significant segmental bone loss (Figure 1). The patient had lost control of his motorcycle while performing “wheelies” and struck a concrete highway divider. His resultant injuries included multiple minor orthopedic fractures and ligament disruptions, including a Gustilo III-B open fracture to the distal left femur. All injuries were evaluated and treated by the initial treating hospital, including external fixation stabilization and two rounds of irrigation and debridement of the open femur fracture.

A thorough history and physical examination revealed that the young patient was in otherwise good health aside from his orthopedic injuries. Distal vascular function to the leg below the level of the open fracture was uninterrupted, and no deficit existed either in the motor or in sensory function of the distal lower extremity. New radiographs and a repeat serial irrigation and debridement upon arrival at our hospital revealed absence of the distal 17 centimeters of metadiaphyseal bone of the left femur. The distal end of the femur was fractured, including extension into the articular surface, but was retained with significant soft tissue attachments. The segment of missing bone included a modest amount of the lateral femoral trochlear articular surface.

The decision on how to best proceed was made after numerous and detailed discussions with both the patient and his family regarding the surgical options of primary amputation of the severely injured leg, or limb salvage. The superior short-term results of primary amputation, as well as a lack of published support for superiority of either technique at two years, were thoroughly discussed [1]. Despite the probability of multiple surgeries over the course of approximately the next year, the attendant risks of each surgery, and the very real possibility of enduring pain and challenging control measures, the patient opted for the limb salvage option. We then set about formulating a surgical plan to provide the best possible outcome to that end.

## 3. Preoperative Planning

The Masquelet technique is a two-stage procedure used to induce growth of new bone in an area of a segmental defect [2]. First published by French orthopaedic pioneer Charles Masquelet whose name the technique bears, the initial step consists of placement of an antibiotic spacer in the area of bone loss and then provisionally fixing both the fracture and spacer with a conventional orthopedic construct, such as plate-and-screw fixation. Over the space of approximately 6 weeks, a foreign body reaction forms a “pseudomembrane” around the cement. The second step consists of exposing this pseudomembrane and then incising it cleanly, exposing the antibiotic spacer underneath. The spacer is then broken up and completely removed along with any attendant screws previously placed into the spacer, leaving the empty pseudomembrane in place at the site of segmental bone loss. This void can then be filled with allograft or autograft bone, or a combination of the two, confined within the limits of the pseudomembrane with the expectation that the space will be filled, in time, with new host bone.

The idea of composite fixation was first advanced by Jeff Mast, MD, as a means in which conventional plate-and-screw fixation could be successfully augmented by supplemental endosteal fixation, creating a stronger and stiffer construct that lateral stabilization alone [3]. The improved stability of composite fixation helps to provide a more stable construct to enhance bony ingrowth into a bone void and, similar to the results of the Masquelet technique, has proven effective at overcoming segmental bone defects.

It was our contention that, just as Dr. Mast's theory of combining endosteal fixation with conventional plate-and-screw fixation resulted in superior stability for a construct compared to conventional techniques, a combination of his technique with the proven efficacy of the Masquelet technique could serve as a powerful combination in the surgical treatment of a particularly challenging case of segmental skeletal defect in a major weightbearing bone. By combining composite fixation with the Masquelet technique, an enduring construct could be created wherein superior stability could be achieved that would allow bone graft to regenerate host bone in such a segmental defect. It was felt that combining these two proven techniques would result in superior results than either used alone.

## 4. Reconstruction Procedures

**Figure 2:**
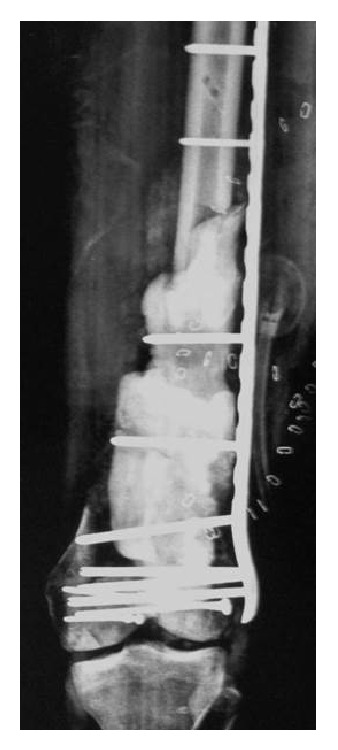
Radiograph following placement of antibiotic cement spacer with lateral distal femoral locking plate fixation.

Once serial irrigation and debridement had resulted in a sterile wound, the defect was prepared for the first step of the Masquelet technique. Attention was first turned to recreating the articular surface of the distal femur. Through direct visualization of the distal femur, screw fixation was used to stabilize the articular surface. Next, an antibiotic-impregnated spacer was fashioned and placed in the area of the segmental femur defect. This was fixed into place using a long lateral femoral locking plate construct (Figure 2). Lastly, the external fixator was removed, the pin sites were curetted, and the femoral musculature was used to cover the femoral construct. A negative pressure wound therapy dressing was reapplied over the exposed muscles and the patient scheduled for routine negative pressure wound therapy dressing changes until the second stage of his reconstructive procedure. Once postoperative antibiotics were complete and pain controlled, the patient was discharged from the hospital with nonweightbearing precautions, VTE prophylaxis, and PRN pain medication for outpatient follow-up.

**Figure 3:**
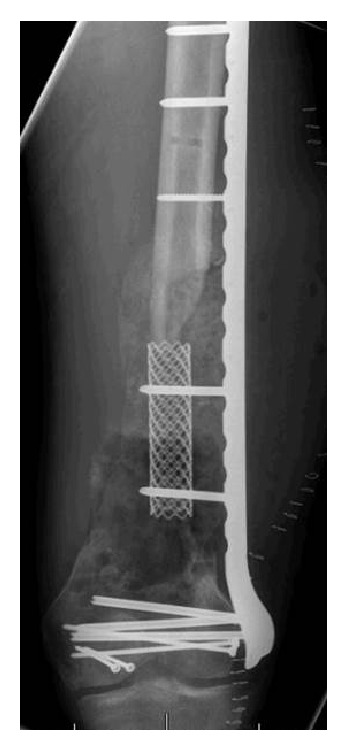
Composite fixation consisting of lateral locking distal femoral plate with endosteal titanium cage augment.

**Figure 4:**
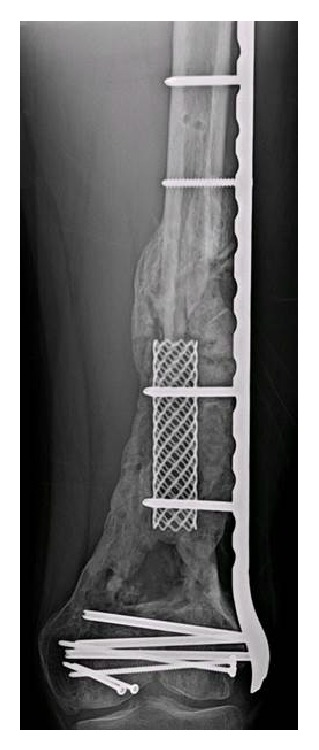
Radiograph demonstrating remarkable regrowth of new bone surrounding the endosteal titanium implant at 8 months post-procedure. Note the contour of the new bone, formed within the pseudocapsule which resulted from the initial antibiotic cement spacer used in the first stage of the Masquelet technique.

Approximately six weeks later, the patient was returned to the operating room for the second stage of his reconstruction procedure. Upon exposure of the distal femur, a robust membrane was encountered with evidence of aggressive callus formation already in place at the site of the antibiotic cement spacer (Figure 4). The pseudomembrane was cleanly incised anteriorly, and the antibiotic spacer was broken up using an osteotome and the fragments were removed. The composite fixation component of the procedure was then introduced by placing a titanium cage fixed into place and extending from the proximal diaphyseal fracture limit to the distal femoral fragment, within the confines of the pseudomembrane (Figure 3). This cage was attached to the lateral plate with screws, providing stabilization for the construct from within as well as the outside of the segmental fracture.

A reamer-irrigator-aspirator unit (RIA, Synthes, West Chester, PA) was next used to aspirate autograft consisting of approximately 60 milligrams of cancellous bone from the contralateral right femur. This autograft bone was combined with an additional 40 milligrams of cancellous allograft bone chips, and the combination was packed within the confines of the pseudomembrane, around the titanium cage. The pseudomembrane was then closed, and the quadriceps musculature was once again brought into place, this time covered by a split-thickness skin graft taken from the contralateral thigh and held in place with a nonadhesive dressing.

## 5. Postoperative Course

The patient was followed in clinic at 2, 4, 6, 8, and 12 weeks postoperatively and monthly thereafter. Gentle, passive range-of-motion exercises were begun under therapist supervision at the fourth postoperative week. Active and active-assist exercises began at 8 weeks. Nonweightbearing status was maintained until the 12th postoperative week, at which time partial weightbearing was allowed, progressing to weightbearing as tolerated by the fourth postoperative month.

As expected, pain control and increased range of motion proved to be two of the patient's greatest hurdles. Pain control was managed with the assistance of Pain Management colleagues, and by the ninth postoperative month, range of motion eventually had increased to a full 180 degrees of extension and 90 degrees of active flexion.

Serial radiographs were monitored for progress of the patient's healing response in the area of the segmental femoral defect. A CT scan was repeated at 9 months to evaluate healing, as well as to more fully appreciate the magnitude of persistent osteochondral defect which measured 60 cm × 40 cm × 10 cm to the lateral aspect of the distal femur, suspected as a continuing pain generator during knee motion.

The patient underwent the final reconstructive procedure at the ninth postoperative month, consisting of allograft reconstruction of the osteochondral defect involving the lateral aspect of the distal femur. The patient underwent this final procedure uneventfully.

## 6. Results

Serial radiographs of the segmental femoral defect demonstrate remarkable, robust filling of the bone void over the course of the six months since the autograft and allograft bone mix was implanted in the second stage of the Masquelet technique. This filling was confirmed on CT during the planning stages for the final osteochondral allograft procedure. The success of this bony reconstitution allowed the patient to return to daily activities, including full weightbearing without need of further assistive devices.

Active and passive range of motion had increased to a full 180 degrees of extension and 90 degrees of flexion by the ninth postoperative month, prior to the final osteoallograft procedure. Return of strength was retarded by persistent pain during the rehabilitative phase of the reconstruction effort. It remains to be seen if this limitation will be overcome by filling of the persistent osteochondral defect. A leg length discrepancy of approximately 3 cm shortening on the operated side persists, which could require the use of a shoe lift.

## 7. Discussion

Segmental defects of the appendicular skeleton, particularly in the weightbearing bones of the lower extremity, are among the most difficult challenges faced by the reconstructive surgeon. The larger the defect, the more creative the surgical plan required to achieve a successful outcome. As a component of limb salvage surgery, they can be some of the most rewarding.

French surgeon Alain-Charles Masquelet developed the two-stage technique that bears his name in the 1980s, describing results of 35 patients with diaphyseal defects ranging from 4 to 25 cm [4]. Before this, the Ilizarov method and vascularized bone transfer were the most common procedures for large diaphyseal bone defects, the former taking months longer for segmental defects, and the latter associated w/unacceptable degrees of graft resorption [5]. But perhaps the most remarkable advantage of the Masquelet technique is that the reconstruction time is independent of the length of the defect [6].

Based on the remarkable results of segmental loss reconstruction, investigation was undertaken to identify the histology of the pseudomembrane formed around the inserted antibiotic spacer utilized in the Masquelet technique. Based on a rabbit model, the induced membranes were hypothesized to contain bone-stimulating factors [7]. Tan et al. undertook to further identify the histology of the pseudomembrane and found it to contain an even greater number of mesenchymal stem cells compared to matched periosteum in a 7-patient cohort, leading the authors to conclude that it was this high concentration of stem cells that proved so successful in reconstruction of large bone defects with the technique [8].

Composite or hybrid fixation entails combined methods of fixation to achieve a construct with superior stability than either single technique alone. Composite fixation can involve fixation within and outside of the cortex of the bone as in our case presentation or other fixation constructs such as internal and external fixation. Advantages include not only superior stiffness in all planes compared to single-phase fixation alone, but composite fixation can be employed as a means of limiting further trauma to an already-traumatized fracture zone. Dekutoski et al. demonstrated the utility of balancing application of stable fixation and limited iatrogenic trauma in their combination of lateral plate and external fixation in six paired specimens involving segmental tibia fractures [9].

Both the Masquelet and composite fixation techniques have documented success in overcoming segmental bone loss independently [7]. Our case of a large segmental defect presented unique challenges for surgical planning of reconstruction for limb salvage and demonstrates the utility of combining the inherent strengths of these two techniques to achieve a successful result. The superior construct stability provided by composite fixation functioned to provide a stable, enduring platform onto which our combination of auto- and allograft bone could thrive within the confines of the pseudomembranous capsule created by use of the Masquelet technique.

Pain control in this patient proved perhaps the most challenging aspect of his postoperative care, and Pain Management consultation was made to optimize his transition from required narcotics in the early postoperative period to nonnarcotic regimens. Even as convincing evidence that nonsteroidal anti-inflammatory pain medication interferes with bone healing is lacking, we chose to avoid NSAIDs in our patient in the early postoperative period [10]. Intravenous Vicodin and morphine were discontinued in favor of morphine sulfate IR, 15 mg every four hours as needed. The Pain Management team continued to follow the patient up and adjust medications accordingly for effective pain control in this challenging patient. Even though narcotic pain medications were necessary early on, an eventual transition to acetaminophen was possible.

Our Infectious Disease team was consulted to direct antibiotic care in addition to our described insertion of the nonbiodegradable antibiotic delivery device spacer described above. The patient had undergone three operative irrigation and debridement procedures at his initial institution prior to his transfer to our hospital, and during none of these previous surgeries, nor any of his subsequent procedures at our institution, did his cultures yield positive results. This was true for intraoperative wound as well as blood and urine cultures. The patient did receive 24 hours of postoperative vancomycin and Zosyn for each of his surgeries at our institution. Upon discharge, he was continued on Cubicin at 4 mg/kg Q 24 hours as well as Invanz 1 gram Q 24 hours, both intravenously, via percutaneous indwelling central catheter for an additional two weeks' period at the recommendations of the Infectious Disease consultant.

Combining the proven techniques of adult reconstruction in creative ways can be used to achieve even greater successes in future cases involving segmental bone loss. Both the Masquelet technique and composite fixation are invaluable tools in overcoming bony voids in the trauma patient. Combining the two is one useful means in addressing complex issues of skeletal reconstruction in this challenging class of patients.
